# Proximity Gettering Design of Hydrocarbon–Molecular–Ion–Implanted Silicon Wafers Using Dark Current Spectroscopy for CMOS Image Sensors

**DOI:** 10.3390/s19092073

**Published:** 2019-05-04

**Authors:** Kazunari Kurita, Takeshi Kadono, Satoshi Shigematsu, Ryo Hirose, Ryosuke Okuyama, Ayumi Onaka-Masada, Hidehiko Okuda, Yoshihiro Koga

**Affiliations:** SUMCO Corporation, 1-52 Kubara, Yamashiro-cho, Imari-shi, Saga 849-4256, Japan; tkadono@sumcosi.com (T.K.); sshigema@sumcosi.com (S.S.); rhirose@sumcosi.com (R.H.); rokuyama@sumcosi.com (R.O.); aonaka@sumcosi.com (A.O.-M.); hokuda@sumcosi.com (H.O.); ykoga4@sumcosi.com (Y.K.)

**Keywords:** gettering, CMOS image sensor, metal impurity, white spot defects, dark current, silicon wafer, dark current spectroscopy

## Abstract

We developed silicon epitaxial wafers with high gettering capability by using hydrocarbon–molecular–ion implantation. These wafers also have the effect of hydrogen passivation on process-induced defects and a barrier to out-diffusion of oxygen of the Czochralski silicon (CZ) substrate bulk during Complementary metal-oxide-semiconductor (CMOS) device fabrication processes. We evaluated the electrical device performance of CMOS image sensor fabricated on this type of wafer by using dark current spectroscopy. We found fewer white spot defects compared with those of intrinsic gettering (IG) silicon wafers. We believe that these hydrocarbon–molecular–ion–implanted silicon epitaxial wafers will improve the device performance of CMOS image sensors.

## 1. Introduction

**Figure 1:**
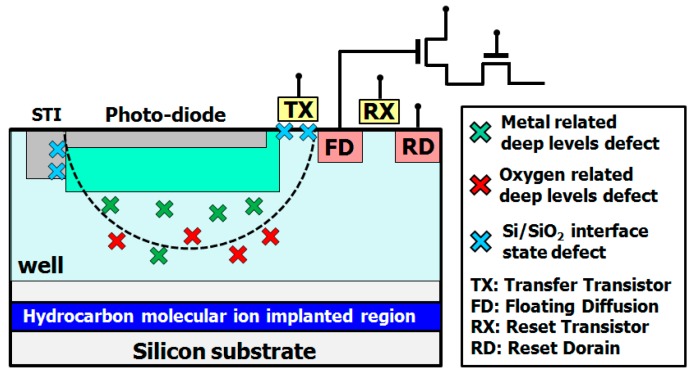
Technological issues of CMOS image sensor fabrication. Modified from Kurita et al. [29], Copyright (2016) The Japan Society of Applied Physics.

Complementary metal-oxide-semiconductor (CMOS) image sensors are widely used in smartphones, smartwatches and tablets computers. Demand from the consumer market for higher sensitivity imaging, wider dynamic range, and higher speed image data processing is driving the development of image sensors with higher performance [1,2]. However, there are serious technical issues with advanced CMOS image sensor manufacturing, as shown in Figure 1.

One issue is metallic impurity contamination in the device’s active region that may occur during fabrication processes such as high-temperature rapid thermal annealing and plasma etching [3,4]. Metallic impurities form deep-energy-level defects in the silicon band gap. These defects in turn strongly affect electrical device parameters such as dark current, white spot defect, recombination lifetime, and transfer gate oxide breakdown voltage [4,5,6,7,8,9]. Thus, CMOS image sensor manufacture requires metallic impurities to be eliminated from the device active region.

The second issue is oxygen out-diffusion from the Czochralski silicon (CZ) silicon substrate to the active region during the device fabrication process [10]. The CZ silicon substrate acquires many oxygen impurities in the silicon crystal bulk during crystal growth. Oxygen impurities form oxygen-related deep energy level defects such as potential barriers or potential pockets in the space charge region of the photo-diode and transfer transistor gate channel [2]. These defects affect the device performance of perfect charge carrier transfer operation such as image lag [1,2].

The third issue is the fixed pattern noise (FIX) and random telegraph signal noise (RTS) induced by interface state traps at Si/SiO_2_ interfaces such as the transfer transistor gate, shallow trench isolation (STI), deep trench isolation (DTI), and local oxidation silicon (LOCOS) [11]. These interface state traps act as generation-recombination (G-R) center that increased FIX and RTS noise. The noise strongly affects the electrical performance parameters of CMOS image sensor devices [12,13].

In the past two decades, semiconductor industrial researchers have tried to resolve the above issues. Metallic impurity contamination is an extremely serious one for advanced CMOS image sensor manufacturing. It has been proposed that gettering techniques can be used to remove this sort of contamination from the device active area during the fabrication process. Here, intrinsic gettering (IG) is widely used in the semiconductor device manufacturing [14,15]. IG forms oxygen precipitates in the silicon crystal bulk that act as gettering sinks during the fabrication process. 

The trend in thermal budgets of CMOS device processes is to use lower temperature and short durations [16]. As a result, it has become extremely difficult for IG to form oxygen precipitates during the advanced CMOS device process. Another gettering technique, extrinsic gettering, uses high energy ion implantation [17,18]. Kuroi et al. reported that implantation of boron under the junction reduces the leakage current of copper contaminated pn-junction compared with a junction that had not been implanted [19]. Here, the high-energy boron implantation forms extended defects such as dislocations and dislocation loops under the junction. These extended defects act as gettering sinks during the CMOS device process. However, this technique induces implantation damage in the top surface region of the wafer [20,21,22,23]. It is very difficult to repair this damage in the case of a low temperature thermal budget, and the defects degrade the device yield and performance of electrical device.

Oxygen out-diffusion is also a critical issue in advanced CMOS image sensor fabrication. Shoyama et al. examined the white spot defect dependence on the initial oxygen concentration in the CZ silicon substrate by using the dark current spectroscopy (DCS) [10]. They found that oxygen impurities out-diffused to the device active region from the CZ silicon substrate during the CMOS device process. The oxygen impurities form deep energy level defects that act as G-R center in the space-charge region and transfer gate channel region. This sort of defect strongly affects the electrical performance parameters of the device. Thus, CMOS image sensor manufacturers have tried to eliminate oxygen impurities from device active region. One way to do so is to use a CZ grown silicon crystal with a low oxygen concentration, which decreases the initial oxygen concentration in the CZ silicon bulk [16]. In this way, it is possible to decrease oxygen diffusion to the device active region during the device fabrication process. However, it is insufficient to control the oxygen out-diffusion to the device active region from CZ grown silicon substrates with a low oxygen concentration.

FIX and RTS noise hinder noise reduction in advanced CMOS image sensors. The most common solution is to use low-temperature hydrogen annealing after the front-end-line (FEOL) process [3]. This annealing treatment can decrease the interface-state traps at Si/SiO_2_ interface defects such as Pb center and E’ centers by being a hydrogen terminated process [24]. However, 3D-stacked CMOS image sensor (3D-CIS) fabrication process often uses atomic layer deposition (ALE) to make multiple stacks of surface deposits [4,25,26]. Multiple dielectric layers are deposited on the device surface before the low-temperature hydrogen annealing treatment. Thus, dielectric layers prevent hydrogen from diffusing into the device active region and most of the hydrogen atoms are trapped in the multiple dielectric layers during the annealing. However, this method does not trap enough hydrogen to prevent process-induced defects passivated by hydrogen. For this reason, manufacturers should develop alternative low-temperature hydrogen annealing for decreasing the interface-state traps in the Si/SiO_2_ interface region [13].

How can we address the above technical issues associated with CMOS image-sensor fabrication? We have undertaken extensive considerations of this question of silicon wafer gettering design and have developed technology such as proximity gettering using hydrocarbon–molecular–ion implantation for advanced CMOS image sensor fabrication processes. We found that a hydrocarbon–molecular–ion–implanted epitaxial silicon wafer has three unique characteristics that improve the electrical device performance of CMOS image sensor [27,28,29]. First, the hydrocarbon–molecular–ion projection range has high gettering capability for metallic impurities [28]. Second, this projection range also has a diffusion barrier effect preventing oxygen impurities from out-diffusing from the CZ silicon grown substrate into the device active region during the CMOS device heat treatment [29]. Third, hydrogen diffuses from the hydrocarbon–molecular–ion projection range into the active region during the CMOS device heat treatment [29].

In this study, we used dark current spectroscopy to compare the metallic impurity gettering capabilities of hydrocarbon–molecular–ion–implanted epitaxial silicon wafers and IG enhanced epitaxial silicon wafers (carbon-doped CZ silicon wafer). Moreover, we studied the dependence of the metal gettering capability on the ion implantation conditions (dose) and epitaxial growth conditions (epitaxial layer thickness).

We found that this novel proximity gettering silicon wafer has higher gettering capability compared with an IG enhanced silicon wafer. Here, we describe the concept of silicon wafer gettering design using hydrocarbon–molecular–ion implantation for advanced CMOS image-sensor fabrication.

## 2. Production of Hydrocarbon–Molecular–Ion–Implanted Epitaxial Silicon Wafer

**Figure 2:**
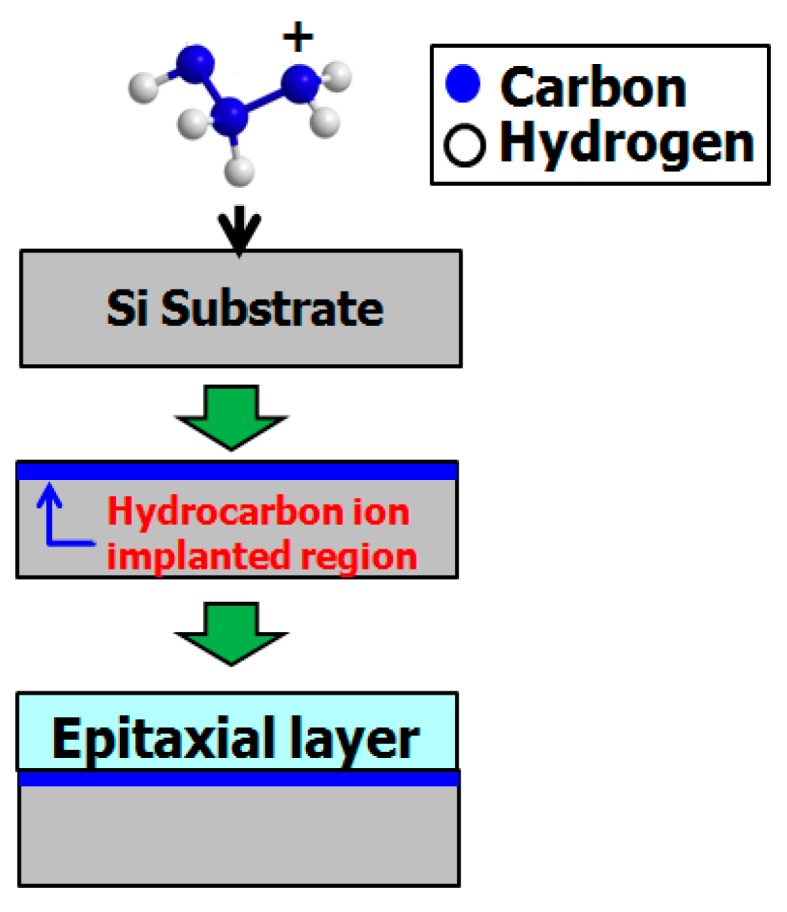
Production of hydrocarbon–molecular–ion–implanted epitaxial silicon wafers. Modified from Kurita et al. [29], Copyright (2016) The Japan Society of Applied Physics.

Figure 2 shows the concept underlying production of hydrocarbon–molecular–ion–implanted epitaxial silicon wafers. First, hydrocarbon molecular ions are generated using the electron impact ionization method [27,28,29]. Second, these ions, such as C_3_H_5_ ion fragments, are implanted in the silicon wafer top-surface region by using a hydrocarbon–molecular–ion implanter (CLARIS, Nissin Ion Equipment, Kyoto, Japan) [30,31,32]. The C_3_H_5_ ion fragments forms as hydrocarbon–molecular–ion projection range after implantation. Finally, an epitaxial layer is deposited on the silicon wafer surface using chemical vapor deposition. The subsequent production does not use an additional heat treatment for re-crystallization of the implantation projection range. Thus, it is very simple for silicon wafer manufacture [27].

**Figure 3:**
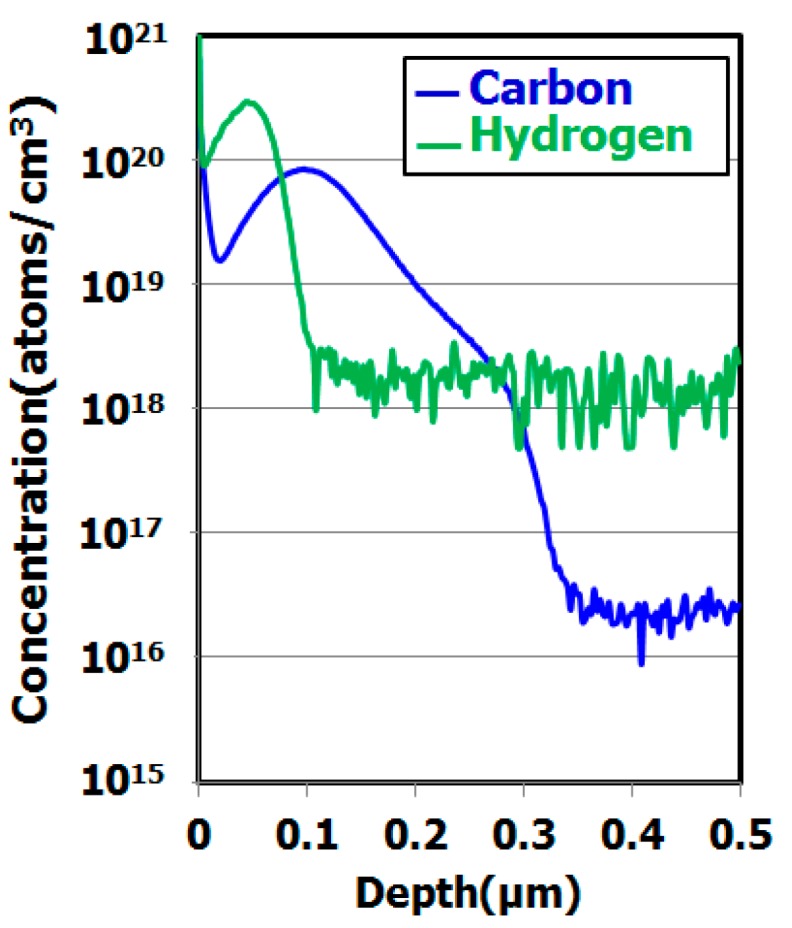
SIMS depth profile of hydrocarbon–molecular–ion–implanted silicon wafers [29]. Copyright (2016) The Japan Society of Applied Physics.

**Figure 4:**
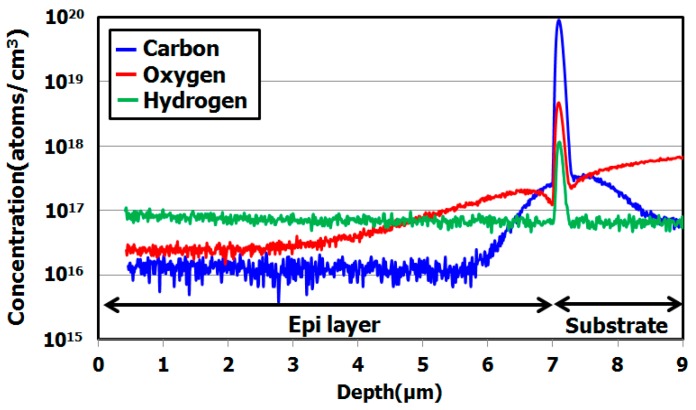
SIMS depth profile of hydrocarbon–molecular–ion–implanted epitaxial silicon wafers. The epitaxial thickness is 7 μm and hydrocarbon dose amount is 1 × 10^15^ cm^−2^. Modified from Kurita et al. [29], Copyright (2016) The Japan Society of Applied Physics.

Figure 3 shows carbon and hydrogen depth profiles in a hydrocarbon–molecular–ion–implanted silicon wafer measured by secondary ion mass spectroscopy (SIMS) [29]. The hydrocarbon molecular ions form a carbon and hydrogen projection range. Figure 4 shows the SIMS depth profile in a hydrocarbon–molecular–ion–implanted silicon wafer after epitaxial growth [29].

**Figure 5:**
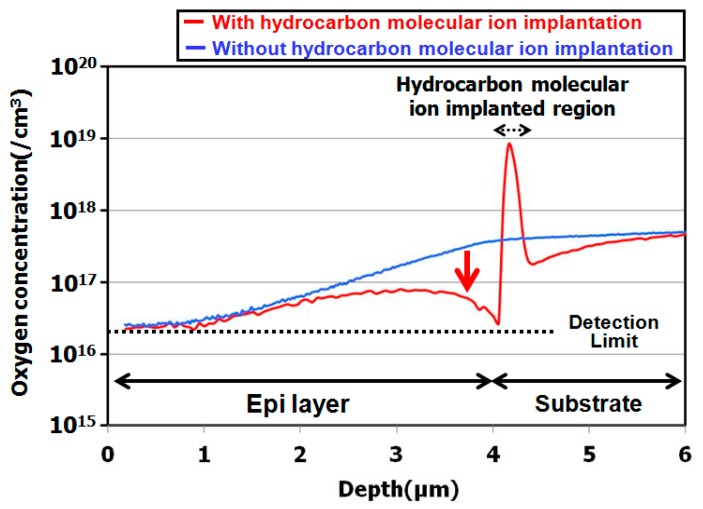
SIMS depth profile of oxygen impurity with and without hydrocarbon–molecular–ion–implanted epitaxial silicon wafers after heat treatment. Modified from Kurita et al. [28], Copyright (2015) The Japan Society of Applied Physics.

The results illustrated in these figures are interesting, because the peak concentration of hydrogen was decreased by the epitaxial growth. The peak concentration of the remaining hydrogen was 10^18^ cm^−3^ in the hydrocarbon–molecular–ion projection range [33,34]. Generally, when monomer-hydrogen is implanted in silicon crystal bulk, it does not remain in the silicon crystal bulk after the epitaxial growth process and instead out-diffuses to the silicon wafer surface and back surface during the growth process. However, in the case of hydrocarbon–molecular implantation, hydrogen is gettered from the hydrocarbon–molecular implantation projected range after the epitaxial growth process. The main reason is that the projection range forms stress and strain fields that act as gettering sinks for hydrogen [33,34]. Furthermore, oxygen impurities out-diffuse into the epitaxial layer from the CZ silicon substrate during the growth process. Oxygen is gettered by the hydrocarbon–molecular implantation projection range [35,36]. Figure 5 shows the depth profile of oxygen impurity with and without hydrocarbon–molecular–ion–implanted epitaxial silicon wafer after heat treatment [28]. This result indicates oxygen impurity out-diffused to the epitaxial layer from CZ silicon substrate during the heat treatment. However, oxygen out-diffusion amount with hydrocarbon–molecular implantation is lower than that without hydrocarbon–molecular implantation in epitaxial layer/substrate interface region. These results indicate that the hydrocarbon–molecular–implanted epitaxial silicon wafer has high gettering capability for light elements such as hydrogen and oxygen.

## 3. Materials and Methods

### 3.1. Sample Preparation

The experimental sample used in this study were 12-inch (100) phosphorus and carbon-doped CZ silicon single crystals that were 750 µm thick. We call them IG enhanced CZ silicon substrates. The dopant concentration was 1 × 10^15^ cm^−3^ for phosphorus and 3 × 10^16^ cm^−3^ for carbon. Their resistivity was 10 Ω cm and their initial oxygen concentration was 1.4 × 10^18^ atoms/cm^3^ (old ASTM). The sample wafers were subjected to C_3_H_5_ implantation with molecular ions at an energy of 80 keV to dose from 1 × 10^14^ to 1 × 10^15^ cm^−2^. The thicknesses of the epitaxial layers deposited on the silicon substrate by chemical vapor deposition were 5 µm and 7 µm. Their resistivity was 10 Ω cm. 

### 3.2. Evaluation Technique for Hydrocarbon–Molecular–Ion–Implanted Silicon Wafers

We measured the metallic impurity concentration in the hydrocarbon–molecular–ion implantation projection range by using secondary ion mass spectroscopy (SIMS). We also measured oxygen precipitation defects in the silicon substrate bulk by using optical microscopy observation method after the CMOS image sensor fabrication process. The structure of hydrocarbon–molecular–ion implantation related defects was analyzed by cross-sectional high-resolution transmission electron microscopy (TEM) and scanning electron microscopy (SEM). We analyzed the impurity distribution mapping of the hydrogen molecular ion implantation projection range at the atomic level by using laser assisted atom probe tomography (APT) [37]. 

### 3.3. Gettering Capability Evaluation Using Dark Current Spectroscopy

We fabricated a CMOS image sensor pixel architecture with a four-transistor-type pinned photo-diode using the CMOS device fabrication process [38,39]. We evaluated the white-spot defects of the image sensor by using dark current spectroscopy (DCS) [40,41,42]. DCS is an extremely powerful metallic contamination analysis tool for charge coupled device (CCDs) and CMOS image sensors. This technique is very similar to deep level transient spectroscopy (DLTS) [43]. In particular, DLTS is able to measure the activation energy and capture cross section for metallic impurities in semiconductor materials using metal Shottkey barrier-junction diodes and pn-junction diodes. Unfortunately, DLTS is not able to measurement the physical parameters of defects in CMOS devices such as metal oxide semiconductor field effect transistor (MOS-FETs) [44]. 

In contrast, DCS can measure metallic-impurity-related defects in conventional CMOS device structure such as MOS-FETs. For this reason, it is often used by manufacturers for testing of CMOS image sensor device performance parameters such as dark current and white-spot defects. Dark current and white-spot defect origins are from deep energy level defects of metallic impurities that form in the photo-diode space charge region during the device fabrication process. McGrath et al. were the first to use DCS to study defect formation and reported that the metallic impurities contaminated CCDs [40]. Domengie et al. used DCS to analyze the dark current spectrum of intentionally doped metallic impurities (Au and W) CMOS image sensor [45]. They observed substantially increased dark current spectrum intensity in metal contaminated CMOS image sensor compared with that of CMOS image sensor without intentional metal contamination. They found that the dark current spectrum intensity strongly depends on the concentration of intentional metallic contamination. Moreover, semiconductor manufacturers have used DCS for metallic impurity contamination analysis of CCD and CMOS image sensor [41,45,46,47,48]. Here, we decided to focus on using DCS to analyze white-spot defects in CMOS image sensors fabricated on an actual production line.

## 4. Results and Discussion

### 4.1. Metallic Impurity Gettering Capability of Hydrocarbon–Molecular–Ion–Implanted Epitaxial Silicon Wafer after Heat Treatment

**Figure 6:**
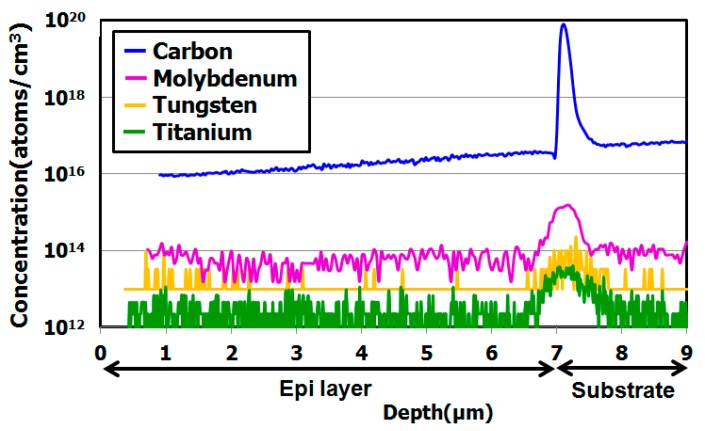
SIMS depth profile of hydrocarbon–molecular–ion–implanted epitaxial silicon wafers after metallic impurity diffusion heat treatment [29]. The epitaxial thickness is 7 µm and hydrocarbon dose amount is 1 × 10^15^ cm^−2^. Modified from Kurita et al. [29], Copyright (2016) The Japan Society of Applied Physics.

Figure 6 shows depth profile of Mo, W and Ti metallic impurities in the implanted epitaxial silicon wafers after they had undergone heat treatment. The intentionally implanted impurities contaminated the surface. The metallic impurity contamination of implantation condition was acceleration energy at 100 keV and dose amount was 1 × 10^11^ cm^−2^ by using monomer ion implanter. After ion implantation and diffusion annealing for metallic impurities, we used SIMS to measure depth profiles of the metallic impurities in the hydrocarbon–molecular implanted projection range. Figure 6 indicates that metallic impurities were gettered by the carbon and hydrogen projection range after the diffusion heat treatment. Thus, this novel silicon wafer has high gettering capability for metallic impurities.

### 4.2. Gettering Capability Dependence on Gettering Methods for Silicon Wafers

**Figure 7:**
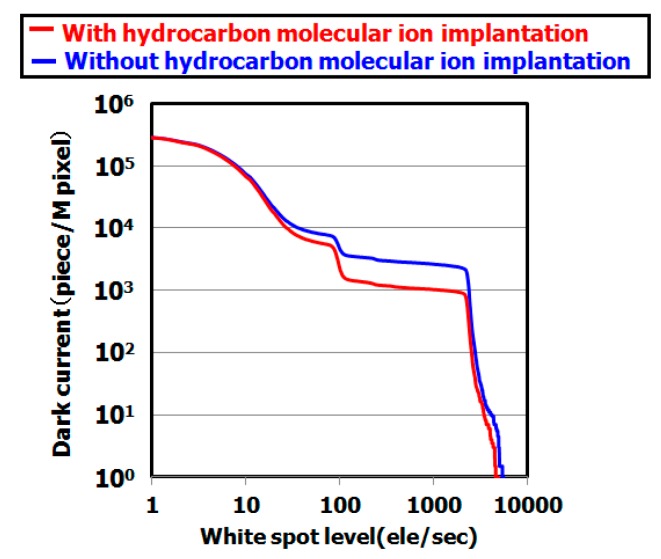
Dark current level after CMOS image sensor fabrication process on epitaxial silicon wafers with and without hydrocarbon–molecular–ion implantation. The epitaxial thickness is 7 µm and hydrocarbon dose amount is 1 × 10^15^ cm^−2^. The dark current levels were measured by dark current spectroscopy under 60 °C.

**Figure 8:**
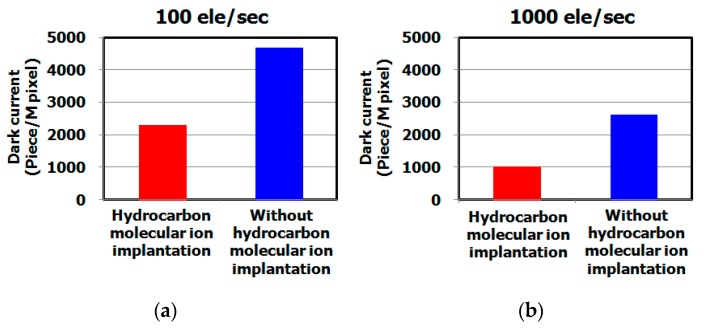
Histograms of dark current level after CMOS image sensor fabrication process on epitaxial silicon wafers with and without hydrocarbon–molecular–ion implantation. The dark current levels were measured by dark current spectroscopy under 60 °C. The epitaxial thickness is 7 μm and hydrocarbon–molecular–ion dose amount is 1 × 10^15^ cm^−2^. (**a**) Number of middle range in white spot level with and without hydrocarbon–molecular–ion implantation, (**b**) Number of large range in white spot level with and without hydrocarbon–molecular–ion implantation.

**Figure 9:**
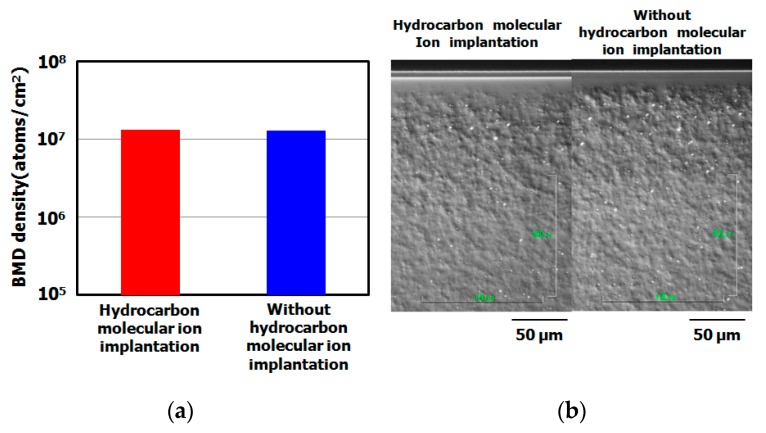
Cross-sectional bulk micro defect (BMD) density determined by optical microscopy observation after CMOS image sensor fabrication process on epitaxial silicon wafers with and without hydrocarbon–molecular–ion implantation. The epitaxial thickness is 7 μm and hydrocarbon–molecular–ion dose amount is 1 × 10^15^ cm^−2^. (**a**) BMD density of silicon wafer substrate with and without hydrogen–molecular–ion implantation after CMOS image sensor fabrication process, (**b**) Optical observation results of BMD depth profile in silicon wafer substrate with and without hydrocarbon–molecular–ion implantation after CMOS image sensor fabrication process.

Figure 7 shows the dark current spectra of CMOS image sensors with and without hydrocarbon–molecular–ion implantation (both sensors used IG enhanced CZ silicon substrate). The dark currents of sensors with hydrocarbon–molecular implantation were substantially fewer than those of the sensor fabricated without the implantation, as shown in Figure 8. Figure 9 shows the results of cross-sectional oxygen precipitation observations made using an optical microscopy measurement. The CMOS image sensors showed no difference in oxygen precipitate defect density after the fabrication process (oxygen precipitate defect: we call them bulk micro defect (BMD)). The IG technique did not affect the dark current reduction phenomenon determines the proximity gettering such as hydrocarbon–molecular–ion implantation. Thus, we supposed that hydrocarbon–molecular–ion implantation enhances the gettering capability compared with the case of the IG enhanced CZ silicon substrate.

### 4.3. Gettering Capability Dependence on Hydrocarbon–Molecular–Ion Implantation Conditions

**Figure 10:**
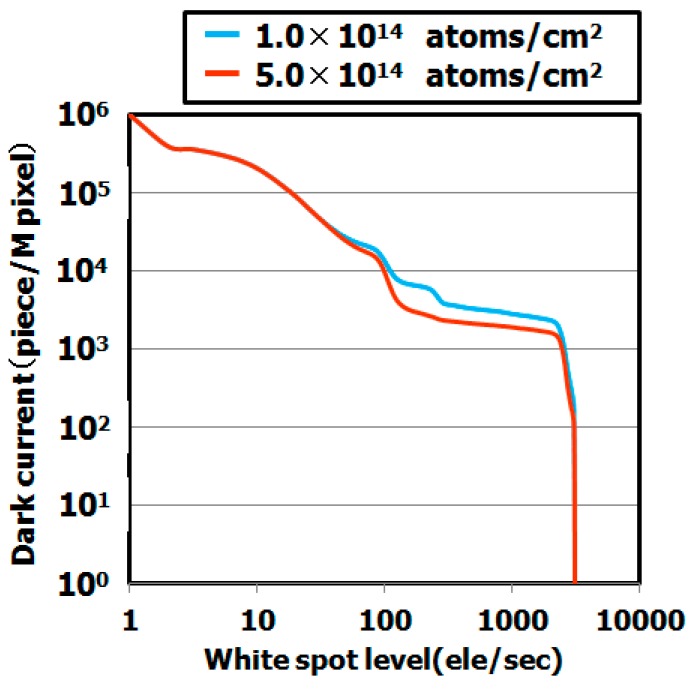
Dark current level of CMOS image sensor depending on hydrocarbon–molecular–ion dose. The dark current levels were measured by dark current spectroscopy under 60 °C. The epitaxial thickness is 5 μm.

**Figure 11:**
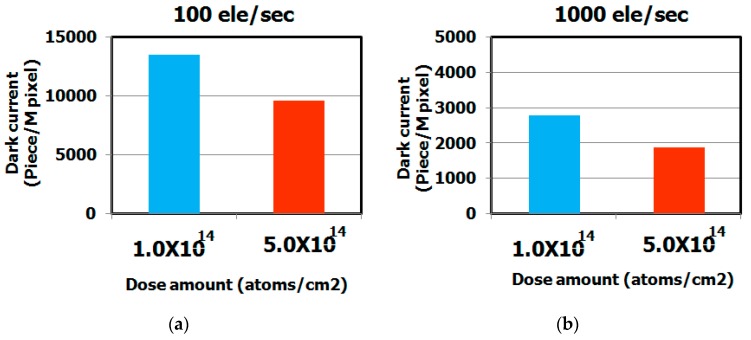
Histograms of dark current level of CMOS image sensor depending on hydrocarbon molecular-ion implantation dose after CMOS image sensor fabrication process. The epitaxial thickness is 5 μm. The dark current levels were measured by dark current spectroscopy under 60 °C. (**a**) Number of middle range in white spot level depends on dose amount, (**b**) Number of large range in white spot level depends on dose amount.

Figure 10 shows the dependence of the dark current spectra on the hydrocarbon molecular dose. These results indicate that the dark current level strongly depends on the dose. The dark current level decreased as the dose increased, as shown in Figure 11. We assume that the dark current levels decreased as a result of the enhanced gettering capability. Our previous study demonstrated that the gettering capability of hydrocarbon–molecular–ion–implanted silicon wafers is strongly correlated with hydrocarbon–molecular–ion implantation dose [29,36].

### 4.4. Gettering Capability Dependence on Epitaxial Growth Conditions (Epitaxial Layer Thickness)

**Figure 12:**
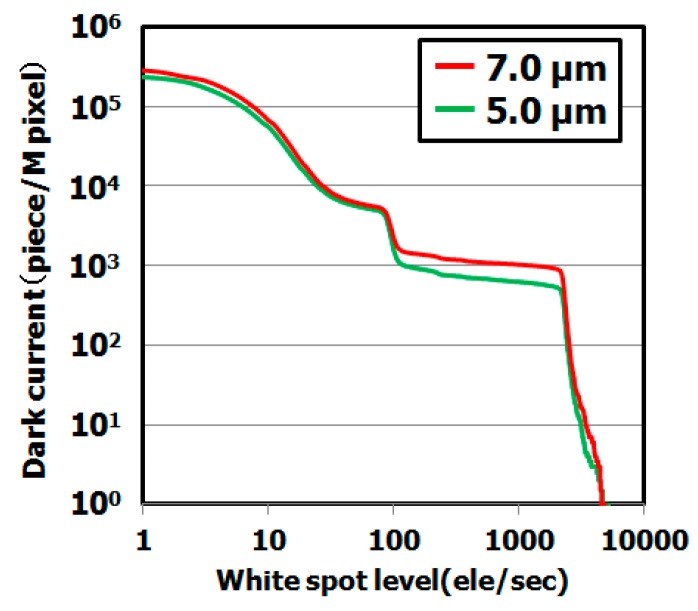
Dark current level of CMOS image sensor depending on epitaxial layer thickness. The dark current levels were measured by dark current spectroscopy under 60 °C. The hydrocarbon–molecular–ion dose amount is 1 × 10^15^ cm^−2^.

**Figure 13:**
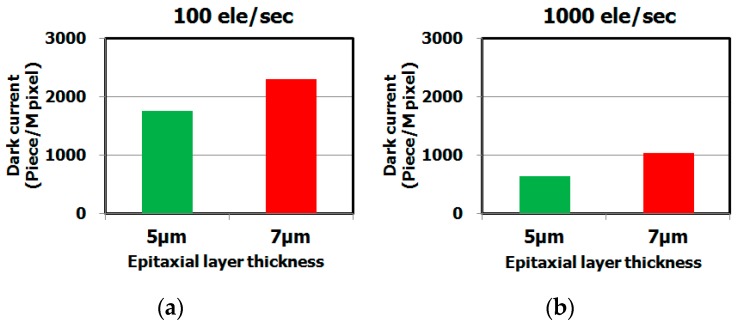
Histograms of dark current level of CMOS image sensor depending on epitaxial layer thickness. The dark current levels were measured by dark current spectroscopy under 60 °C. The hydrocarbon–molecular–ion dose amount is 1 × 10^15^ cm^−2^. (**a**) Number of middle range in white spot level depends on epitaxial layer thickness, (**b**) Number of large range in white spot level depends on epitaxial layer thickness.

Figure 12 shows the dependence of the dark current spectra for different epitaxial layer thicknesses (thickness: 5 μm and 7 μm). The dark current level of the sample with the 5 μm epitaxial layer was lower than that of the sample with the 7 μm epitaxial layer. This result indicates the dark current level strongly depends on the epitaxial layer thickness, as shown in Figure 13. We assume that the metallic impurity gettering capability depends on position of the gettering sinks under the active region.

### 4.5. TEM and APT Observation Results of Hydrocarbon–Molecular–Ion–Implanted Epitaxial Silicon Wafer after CMOS Image Sensor Fabrication Process

**Figure 14:**
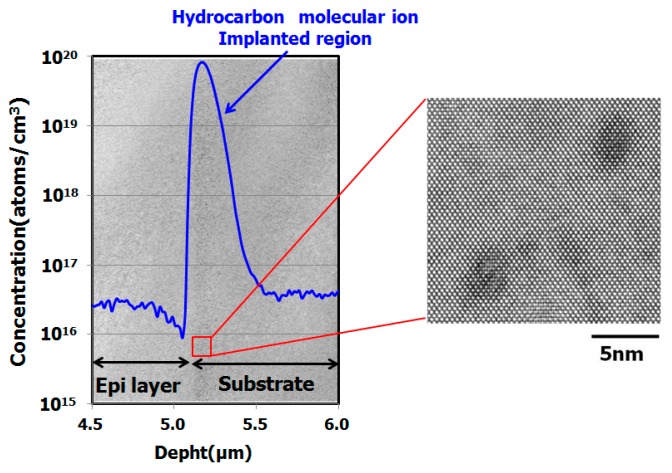
Cross-sectional TEM observation and SIMS depth profile of carbon in hydrocarbon–molecular–ion implantation projection range after CMOS image sensor fabrication process [36].

Figure 14 shows the results of cross-sectional TEM observation of a hydrocarbon–molecular–ion–implanted epitaxial silicon wafer after the sensor fabrication process [36]. We found the implantation related defects in hydrocarbon–molecular–ion implantation projection range. These defects had a density of approximately 1 × 10^6^ cm^−3^ and a size of 5 nm. No secondary extended defects such as dislocations and dislocation loops were observed in the hydrocarbon–molecular–ion–implantation projection range.

**Figure 15:**
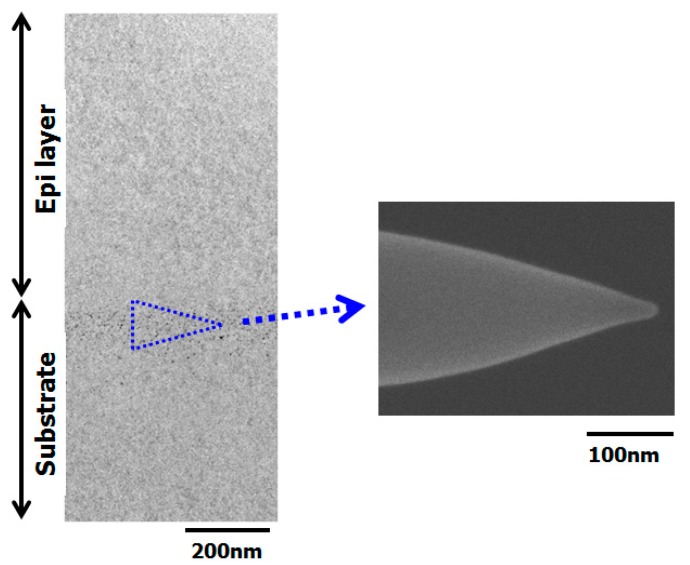
Cross-sectional SEM observation result for atom probe tomography (APT) needle-shaped specimen prepared by field ion beam from hydrocarbon–molecular–ion–implanted epitaxial silicon wafers after CMOS image sensor fabrication process [36].

**Figure 16:**
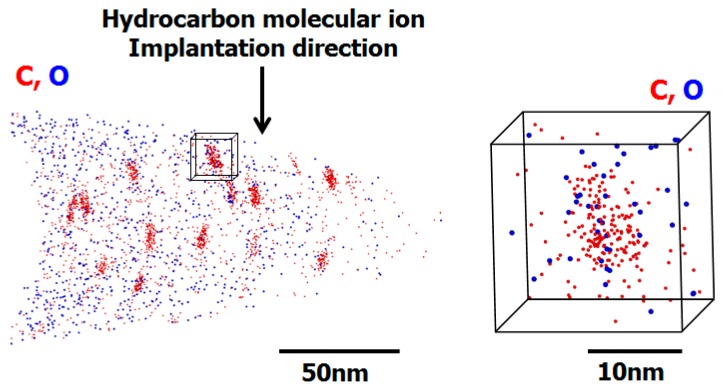
Atom probe tomography map of hydrocarbon–molecular–ion implantation projection range after CMOS image sensor fabrication process [36].

Figure 15 shows the results of a cross-sectional SEM observation of an APT needle-shaped specimen prepared using a field ion beams from a hydrocarbon–molecular–ion–implanted epitaxial silicon wafer after the CMOS image sensor fabrication process [36]. After preparing the specimen, we used APT to measure the three-dimensional impurity distribution in the hydrocarbon–molecular–ion–implanted epitaxial silicon wafer after CMOS image sensor fabrication process. Figure 16 shows the APT map of the hydrocarbon–molecular–ion implantation projection range [36]. The data indicate that the carbon atoms agglomerated into carbon cluster complexes during the CMOS image sensor fabrication process. Moreover, oxygen atoms became segregated from carbon complexes during the CMOS image sensor heat treatment.

### 4.6. Metallic Impurity Gettering Mechanism of Hydrocarbon–Molecular–Ion–Implanted Silicon Wafer

Figure 7 indicates that the hydrocarbon–molecular–ion–implanted silicon wafer can substantially decrease white-spot counts compared with a conventional silicon wafer (IG enhanced silicon wafer). These results show that these wafers have high gettering capability for metallic impurities during CMOS device fabrication processes. 

Why do hydrocarbon–molecular–ion–implanted silicon wafers have such high gettering capability during the CMOS device fabrication processes?

As an answer, we describe below two possible gettering mechanism.

#### 4.6.1. Relaxation-Induced Gettering Mechanism

High energy ion implantation induces defects such as dislocations and dislocation loops [19,49,50]. These defects form strain and stress field in silicon crystal bulk. Metallic impurities become captured by these extended defects. A previous study reported that high energy ion implantation effectively form gettering sinks for metallic impurities. This phenomenon is explained by the Cottrell effect, in which the solubility of an impurity atom will be greater in the vicinity of secondary extended defects such as dislocations and dislocation loops [17,18,19]. However, in our experiment, we did not observe any secondary extended defects in the hydrocarbon–molecular–ion implantation projection range after the fabrication process as shown in Figure 14. Thus, a relaxation-induced gettering mechanism cannot account for our experimental results.

#### 4.6.2. Segregate-Induced Gettering Mechanism

In segregate-induced gettering, the solid solubility of metallic impurities in the gettering sinks increases substantially more than in silicon crystal bulk without gettering sinks [51]. Figure 6 indicates that the metallic impurity concentration in the hydrocarbon–molecular–ion projection range is higher than that in the solid solubility solution of silicon crystal bulk. Thus, the experimental results indicate that the hydrocarbon–molecular–ion implantation forms segregate-induced gettering sinks in the implantation projection range.

#### 4.6.3. Origin of Gettering Sinks in Hydrocarbon–Molecular–Ion Implanted Projection Range

What is the origin of gettering sinks in the hydrocarbon–molecular–ion projection range?

We observed the hydrocarbon–molecular–ion projection range after CMOS image sensor fabrication process by using APT [48]. The results indicate that the defects form carbon oxygen agglomerations such as carbon complexes in the implantation projection range. We analyzed the APT mapping data of carbon complexes to determine the defect density and size by using IVAS data analysis software (CAMECA, Fitchburg, WI, USA) [36]. The carbon complex density was 1 × 10^16^ cm^−3^ and the size was 5 nm [29,36,52]. This means the APT mapping data and TEM observed show the same hydrocarbon–molecular–ion implanted related defects such as carbon complexes in the implantation range after the CMOS image sensor fabrication process (See Figure 14). 

Why do the gettering sinks of carbon complexes in the ion implantation projection range work for metallic impurities?

We suppose that these gettering sinks strongly interact with metallic impurities in the hydrocarbon–molecular–ion implantation projection range.

Shirasawa et al. considered these issues from the view point of theoretical solid-state physics [53,54,55]. They conducted a first-principles calculation to determine the binding energies of metallic impurities and carbon complexes with intrinsic point defects. Their calculation indicates the cause of the effectiveness of those defects in hydrocarbon–molecular–ion implantation gettering sinks. They indicate that the origin of the metallic impurity gettering sinks consists of interstitial carbon and intrinsic point-defects complexes (carbon self-interstitial cluster, vacancy–oxygen pairs and vacancy–hydrogen pairs). Moreover, our previous study demonstrated that the gettering behavior of hydrogen in the projection range of hydrocarbon–molecular–ion implantation after epitaxial growth can be calculated using technology computer aided design (TCAD) incorporating a reaction model in which hydrogen binds to a carbon and silicon self-interstitial cluster (Cs-I) [35,36]. The calculation indicated that a Cs-I is an extremely effective gettering sink for metallic impurities in the hydrocarbon–molecular–ion implantation projection range.

Here, we propose that the hydrocarbon–molecular–ion implantation projection range must be formed in configurations such as carbon complexes (Cs-I), vacancy–oxygen pairs and vacancy–hydrogen pairs for effective gettering sinks to form. We used APT for analyzing implantation-related defects in the implantation projection range after CMOS image sensor fabrication process (see Figure 16). We found that the observed carbon complexes are gettering sinks in the hydrocarbon–molecular–ion implantation projection range. We thus believe that the origin of the segregated-induced gettering sinks are carbon complexes (Cs-I), vacancy–oxygen pairs and vacancy–hydrogen pairs in a hydrocarbon–molecular–ion implantation projection range.

## 5. Gettering Technology Design for Back-Side-Illuminated CMOS Image Sensors

What is the best choice of gettering technique for back-side-illuminated CMOS image sensors?

**Figure 17:**
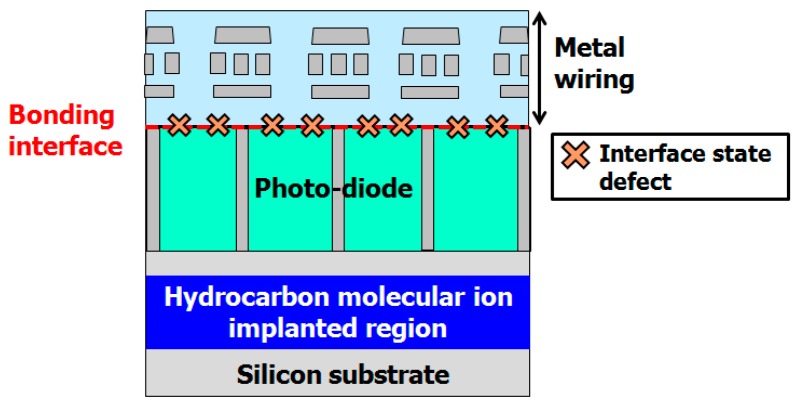
Gettering technology design of back-side-illuminated CMOS image sensors.

Back-side-illuminated CMOS image sensors (BSI) are being manufactured for the consumer mobile phone market, because their quantum efficiency is higher than that of front illuminated CMOS image sensors [25,26]. However, there are serious problems in the BSI fabrication process, as shown in Figure 17. One is metallic impurity contamination during the thin-wafer fabrication process; the thickness of a BSI silicon wafer is less than 10 μm. That is, only the epitaxial layer remains after backside-grinding and chemical mechanical polishing (CMP) completely remove the CZ silicon wafer substrate. The gettering sinks are thus eliminated by the BSI device fabrication process. Lee et al. examined copper contamination of the backside grinding process used in thin MOS-FET device fabrication. They made evaluated by MOS capacitance generation-recombination lifetime measurements and found that the copper impurities in-diffuse into the device active region during the backside grinding process and CMP process [9,56,57,58]. Copper impurities then form deep-energy level defects in the silicon band-gap. The defects act as G-R centers. Thus, they degrade the generation-recombination lifetime and device electrical performance. 

Another important issue is interface-state defects induced by direct wafer bonding in the BSI fabrication process [59]. The direct wafer bonding technique uses a high-energy ion beam irradiation for surface activation. Thus, the wafer surface forms ion-beam-related defects after ion beam irradiation. These defects affect the time dependent dielectric breakdown voltage (TDDB) and RTS noise. CMOS image sensor manufacturers require for a way of dealing with these issues. We propose using hydrocarbon–molecular–ion–implanted epitaxial silicon wafer [60,61]. 

There are three reasons that using hydrocarbon–molecular–ion–implanted epitaxial silicon wafer is the solution of the above issue. 

First, Yamaguchi et al. reported that hydrocarbon–molecular–implanted silicon wafer can achieve a decrease of metallic impurities related dark current and that of interface state defect related dark current in deep trench isolation (DTI) or Si/SiO_2_ interface region using CMOS image sensor fabrication [62,63,64] (Yamaguchi et al. called this wafer “carbon complexes”). Their experimental results indicate that the interface state defect passivated by hydrocarbon (mainly hydrogen). They understand that the hydrogen out-diffused to the DTI or Si/SiO_2_ interface region from hydrocarbon implantation projection range during the CMOS device fabrication process. Out-diffused hydrogen will be adsorbed the DTI or Si/SiO_2_ interface structure defects such as Pb and E’ centers. As a result, this wafer can decrease of DTI or Si/SiO_2_ interface related defects during the CMOS device fabrication process.

Second, our previous study demonstrated that pn-junction leakage current of pn-junction diode dramatically decreased by combination of both hydrocarbon–molecular implantation and surface activated wafer bonding (SAB) techniques compared to without SAB [65,66]. The pn-junction leakage current determined some factors such as metallic impurity contamination in space-charge region and interface state defect in device isolation region. With SAB wafer has two effective gettering sinks under the epitaxial silicon layer. One is hydrocarbon–molecular-ion projection range, and the other is SAB bonding regions. Hydrogen storages in hydrocarbon–molecular–ion projection range. This hydrogen out diffuses to the isolation region during the device heat treatment. The isolation-related interface state defects were passivated by hydrogen. Moreover, the SAB bonding region formed stress and strain field [67,68,69]. This field can act as effective getting sinks during the device fabrication process. Our experimental results indicate that the with SAB wafer can improve the pn-junction leakage current.

Third, we demonstrated that the hydrogen out-diffused to silicon epitaxial layer (device active region) from hydrogen storage in hydrocarbon–molecular–ion projection range during the heat treatment [35,65,66]. This hydrogen of out diffusion amount is 10^12^ to 10^13^ cm^−2^ measured by SIMS after heat treatment [70]. It is well known that Si/SiO_2_ interface state density in MOS transistor device is approximately 10^10^ to 10 ^11^ cm^−2^ [71]. The hydrogen amount of hydrocarbon–implanted silicon wafer is two or three orders of magnitude higher than the typical Si/SiO_2_ interface state density in MOS transistor device. Thus, we believe that the Si/SiO_2_ interface state defect passivated by diffused hydrogen from hydrocarbon–molecular–ion projection range during heat treatment.

Our proposal gettering design concept for silicon wafer production leaves intact gettering sinks in the epitaxial layer after backside grinding and the CMP process in BSI fabrication. We previously reported that the metallic impurity gettering capability of this wafer was higher than that in the CZ silicon substrate using APT [60,61]. Because the gettering capability depends on the depth profile of gettering sinks in the silicon epitaxial layer.

Regarding the TDDB and RTS noise issues, CMOS image sensor manufacturers use a low-temperature hydrogen sintering treatment to decrease interface-state defects by hydrogen passivation. However, it is extremely difficult for hydrogen to diffuse to the wafer bonding interface after the metallization process. The metal electrode and die-electrode film act as a hydrogen diffusion barrier during the hydrogen sintering treatment. Thus, the hydrogen does not diffuse the wafer bonding interface. However, our wafer stores hydrogen in the hydrocarbon–molecular–ion implantation projection range of the epitaxial layer [35,65,66]. Hydrogen diffuses into the bonding interface region during the heat treatment [65,66]. This hydrogen diffuses to the wafer bonding interface during the BSI fabrication process. We suppose that wafer-bonding-interface-state defects are passivated by this hydrogen. Our novel silicon wafer can decrease interface state defect density during the BSI fabrication process. Therefore, we think that it is a solution to the above technical problems.

## 6. Conclusions

CMOS image sensors are ubiquitous devices and demand from the consumer market has driven the rise in performance of these sensors. However, technical issues such as metallic impurity contamination during device fabrication have hindered their manufacture. Here, we developed a metallic impurity gettering technique that uses hydrocarbon–molecular–ion–implanted epitaxial silicon wafers. This novel silicon wafer technology can dramatically decrease dark current during the sensor fabrication process. We conclude that silicon wafers made with this technology have higher gettering capability compared with conventional epitaxial silicon wafers (IG enhanced epitaxial silicon wafers). We believe that this novel technology will improve CMOS image sensor performance.

## 7. Patents

1. Kadono, T.; Kurita, K. Method of producing semiconductor epitaxial wafer, semiconductor epitaxial wafer, and method of producing solid-state image sensing device. Japan Patent 5,673,811, 9 January 2015.
2. Kadono, T.; Kurita, K. Method of producing semiconductor epitaxial wafer, semiconductor epitaxial wafer, and method of producing solid-state image sensing device. Japan Patent 5,799,935, 4 September 2015.
3. Kadono, T.; Kurita, K. Method of producing semiconductor epitaxial wafer, semiconductor epitaxial wafer, and method of producing solid-state image sensing device. Japan Patent 5,799,936, 4 September 2015.
4. Kadono, T. Method of producing epitaxial silicon wafer, epitaxial silicon wafer, and method of producing solid-state image sensing device. Japan Patent 5,776,669, 9 September 2015.
5. Kadono, T. Method of producing epitaxial silicon wafer, epitaxial silicon wafer, and method of producing solid-state image sensing device. Japan Patent 5,776,670, 17 July 2015.
6. Kadono, T. Method of producing semiconductor epitaxial wafer, semiconductor epitaxial wafer, and method of producing solid-state image sensing device. U.S. Patent 9,224,601,29 December 2015.
7. Kadono, T. Semiconductor epitaxial wafer. U.S. Patent 9,397,172, 19 July 2016.
8. Kadono, T.; Kurita, K. Method of producing semiconductor epitaxial wafer, semiconductor epitaxial wafer, and method of producing solid-state image sensing device. U.S. Patent 9,496,139, 15 November 2016.
9. Iwanaga, T.; Kurita, K.; Kadono, T. Method of producing epitaxial silicon wafer and epitaxial silicon wafer. Japan Patent 6,056,772, 16 December 2016.
10. Kadono, T.; Kurita, K. Method of producing epitaxial silicon wafer, epitaxial silicon wafer, and method of producing solid-state image sensing device. Japan Patent 6,107,068, 17 March 2017.
11. Kadono, T.; Kurita, K. Method of producing semiconductor epitaxial wafer, semiconductor epitaxial wafer, and method of producing solid-state image sensing device. Japan Patent 6,221,928, 13 October 2017.
12. Kadono, T.; Kurita, K. Method of producing bonded silicon wafer and bonded silicon wafer. Japan Patent 6,229,258, 27 October 2017.
13. Shigematsu, S.; Okuyama, R.; Kurita, K. Gettering capability evaluation mehod and epitaxial silicon wafer. Japan Patent 6,327,393, 27 April 2017.
14. Kadono, T.; Kurita, K. Method of producing semiconductor epitaxial wafer, semiconductor epitaxial wafer, and method of producing solid-state image sensing device. Japan Patent 6,278,592, 26 January 2018.
15. Kadono, T.; Kurita, K. Method of producing semiconductor epitaxial wafer, semiconductor epitaxial wafer, and method of producing solid-state image sensing device. Japan Patent 6,289,805, 16 February 2018.
16. Kadono, T.; Kurita, K. Method of producing bonded silicon wafer and bonded silicon wafer. Japan Patent 6,265,291, 15 January 2018.
17. Kadono, T.; Kurita, K. Method of producing semiconductor epitaxial wafer, semiconductor epitaxial wafer, and method of producing solid-state image sensing device. Japan Patent 6,278,591, 26 January 2018.
18. Kadono, T.; Kurita, K. Method of producing epitaxial silicon wafer, epitaxial silicon wafer, and method of producing solid-state image sensing device. Japan Patent 6,280,301, 26 January 2018.
19. Kadono, T.; Kurita, K. Method of producing epitaxial silicon wafer, epitaxial silicon wafer, and method of producing solid-state image sensing device. Japan Patent 6,361,779, 6 July 2018.
20. Iwanaga, T.; Kurita, K.; Kadono, T. Epitaxial wafer manufacturing method and epitaxial wafer. U.S. Patent 10,062,569, 28 August 2018.
21. Iwanaga, T.; Kurita, K. Method of producing epitaxial silicon wafer. Japan Patent 6,413,238, 12 October 2018.
22. Kadono, T.; Kurita, K. Method of producing semiconductor epitaxial wafer and method of producing solid-state image sensing device. Japan Patent 6,459,948, 11 January 2019.
23. Kadono, T.; Kurita, K. Method of producing semiconductor epitaxial wafer and method of producing solid-state image sensing device. Japan Patent 6,485,315, 1 March 2019.
